# Association of blood progesterone levels on trigger day and low oocyte retrieval in cases with polycystic ovary syndrome undergoing in-vitro fertilization: A prospective cohort study

**DOI:** 10.18502/ijrm.v23i5.19266

**Published:** 2025-07-29

**Authors:** Nahid Bahrami, Ashraf Moini, Ladan Kashani, Mahshad Khodarahmian

**Affiliations:** ^1^Department of Obstetrics and Gynecology, School of Medicine, Arash Women's Hospital, Vali-E-Asr Reproductive Health Research Center, Tehran University of Medical Sciences, Tehran, Iran.; ^2^Department of Obstetrics and Gynecology, School of Medicine, Arash Women's Hospital, Tehran University of Medical Sciences, Tehran, Iran.; ^3^Department of Endocrinology and Female Infertility, Reproductive Biomedicine Research Center, Royan Institute for Reproductive Biomedicine, ACECR, Tehran, Iran.; ^4^Breast Disease Research Center (BDRC), Tehran University of Medical Sciences, Tehran, Iran.; ^5^Department of Anatomical Science, Tehran University of Medical Sciences, Tehran, Iran.

**Keywords:** Fertilization in-vitro, Oocyte, Polycystic ovary syndrome, Progesterone, Estradiol.

## Abstract

**Background:**

The number of follicles aspirated during intracytoplasmic sperm injection (ICSI)/in vitro fertilization (IVF) treatment does not always match the number of oocytes recovered.

**Objective:**

This study aimed to assess the oocyte retrieval rate (ORR) distribution data and investigate the risk factors for low ORR in polycystic ovary syndrome (PCOS) cases undergoing IVF/ICSI.

**Materials and Methods:**

This prospective cohort study was conducted on 140 women aged between 18 and 40 yr with PCOS who were referred to Arash hospital in Tehran, Iran for ICSI/IVF treatment from March to November 2024. The ratio of obtained oocytes to follicles ( ≥ 17 mm) on the trigger day was used to determine the OPR. 140 women were split into 2 groups: one with a low ORR and one with a normal ORR, each separated by one standard deviation from the ORR mean.

**Results:**

No significant difference was observed between the low and normal ORR groups for progesterone levels. A statistically significant difference was observed in terms of estradiol/follicle ratio above 17, number of follicles above 17, and number of retrieved eggs between the low and normal ORR groups. Logistic regression analysis showed that serum estradiol/follicle ratio ( ≥ 17 mm) with (OR = 0.96, 95% CI [0.94–0.98], p = 0.001) was a factor affecting low ORR.

**Conclusion:**

Low ORR, which results in fewer embryos and more cycle cancellations, may be caused by low progesterone levels on the trigger day, low estradiol levels/follicles ( ≥ 17 mm), and the use of the progestin-primed regimen in PCOS cases.

## 1. Introduction

Oocyte retrieval is an important step in intracytoplasmic sperm injection (ICSI) and in vitro fertilization (IVF) (1). Numerous studies have examined the ovarian response to gonadotropin stimulation; however, comparatively less attention has been paid to the phenomenon called as an empty follicle, which occurs when oocytes are not recovered from a dominant follicle with normal ultrasound function (1–2). A disease known as empty follicle syndrome occurs when oocyte retrieval during ovarian stimulation is impossible, even in the presence of adequate follicle development and adequate levels of the hormone estradiol (3). Failure to extract oocytes is rare and undesirable in cycles with only 1 or 2 dominant follicles (4). However, oocytes from patients with a high pre-ovulation follicle count were sometimes much smaller than those from the pierced follicles (5).

Although some of them were able to become pregnant, it is unclear if this type of outcome was due to the operator's error or the oocyte's aberrant growth in the follicle (6). The likelihood of an oocyte being produced by a mature follicle is 80% (7). Therefore, after piercing 5–6 follicles, a woman should theoretically have a less than 1% chance of not getting an oocyte (8). Assume that a minor quantity of oocyte loss is acceptable and that the stimulating medication is taken as prescribed. In that scenario, as more follicles are punctured, the chance of failure or inadequate oocyte retrieval should decrease. The number of recovered oocytes divided by the number of follicles was known as oocyte retrieval rate (ORR).

Participants with polycystic ovary syndrome (PCOS) are good candidates for investigating low oocyte retrieval. They have more follicles before ovulation and a high expected oocyte count when the ovaries respond normally to gonadotropins (9–10).

Considering this issue and the lack of studies on risk factors affecting low oocyte recovery in cases with PCOS undergoing IVF, it seems logical to review, design, and implement a study in this field. Therefore, this study was conducted to investigate the relationship between blood progesterone levels on the trigger day and low oocyte retrieval in cases with PCOS undergoing IVF.

## 2. Materials and Methods 

From March to November 2024, 140 women with PCOS, ages 18–40, who were sent to Arash hospital in Tehran, Iran, as IVF candidates, participated in this prospective cohort research. Women with at least 2 of the 3 conditions -irregular menstruation, hyperandrogenism, and ultrasound confirmation of PCOS, the presence of follicles ≥ 12 with a size of 2–9 mm in at least one ovary, or the volume of the ovary was > 10 cm^2^ in at least one ovary- were eligible for the Rotterdam criteria-based PCOS diagnosis (11).

Cases with congenital adrenal hyperplasia, androgen-secreting tumors, Cushing's syndrome, thyroid dysfunction, hyperprolactinemia, body mass index (BMI) > 30, first-time PCOS diagnosis, cancellation of ovarian stimulation cycle, history of endometriosis, history of ovarian surgery, and cases with intramural fibroids > 3 cm were excluded from the study. The Rotterdam criteria-based PCOS cases and IVF candidates were included in the study. On days 1–3 of the menstrual cycle, participants with PCOS were referred to the hospital, where the infertility specialist performed a vaginal ultrasound of the ovaries. The participants received 250 mg of gonadotropin-releasing hormone (GnRH) antagonist daily as part of the typical routine antagonist treatment. After 5–6 days, they were given recombinant follicle-stimulating hormone (FSH).

Based on women's age and BMI, the initial dose of r-FSH was 150–250 IU. This was subsequently modified based on the estimated ovarian utilizing vaginal-trans scans to ensure that at least 2–3 follicles with a diameter of 18–20 mm had formed. The antagonist protocol employed 0.2 mg of GnRH ampoule agonist (0.1 Decapeptyl) to induce ovulation. 36–37 hr after stimulation, all oocytes with a diameter of at least 17 mm were punctured and aspirated using a 17G needle at a pressure of 120 mmHg. The follicular fluid was immediately examined under a stereomicroscope with a 6–10x magnification, and the quantity of oocytes in cumulus complexes with healthy zona pellucida was recorded.

The ratio of the number of follicles with a diameter of at least 17 mm on the first day of the trigger to the number of oocytes collected was known as the oocyte retrieval rate, or ORR.

With a restriction of one standard deviation from the mean ORR value that was logarithmically converted, participants were split into 2 groups: one for low ORR and one for normal ORR.

Because the study population was tiny, we decided not to use a cutoff point. Although this difference was not statistically significant, the low ORR group had lower progesterone levels on the trigger day than the normal ORR group. The limited size of the research population could be the cause of this. Double trigger for oocyte maturation can be considered when comparing the number of oocytes extracted to the typical number and size of follicles in the low ORR group, as well as the lower ratio of estradiol to follicles with a size of 17 mm or greater compared to the normal ORR group. A low dosage of the human chorionic gonadotropin (hCG) hormone ampoule is also advised for PCOS patients.

A blood draw to measure blood levels of progesterone and estradiol was the only intervention carried out on the trigger day. All women who were referred for IVF therapy underwent the remaining interventions on a regular basis. On the trigger day, the individuals' levels of progesterone and estradiol were assessed, and they were split into 2 groups: the exposure group, which had high progesterone, and the non-exposure group, which had low progesterone. The results of the trial were measured, and participants were monitored until IVF was done. Furthermore, the Rotterdam criteria for PCOS diagnosis and IVF/ICSI applicants were eligible to participate in the study if they met the following requirements: women between the ages of 18 and 40 were eligible to participate in their first or subsequent IVF cycles.

Male factor infertility, tubal blockage, hydrosalpinx, mild endometriosis, poor response to ovulation stimulation, and unsuccessful intrauterine insemination are some of the causes of infertility.

Following the antagonist protocol, patients were given 250 µg of GnRH antagonist (Merc, Actoverco) every day. They were given recombinant FSH (Cinnal-F, produced by CinnaGen), 5–6 days later. The antagonist protocol used 0.2 mg of GnRH amp agonist (0.1 Decapeptyl, produced by Varian Pharmed Co.) to induce ovulation. One standard deviation was subtracted from the average ORR in this study, which was considered to be 50%, as a division criterion. Therefore, the normal group was characterized as women with an ORR > 50, while women with an ORR of < 50 (or 0.5) were deemed to have a low ORR. Potential confounders like BMI, antral follicle count (AFC), and past ovarian stimulation history were not taken into account by the participants.

### Sample size

The method of calculating the sample size and its number was based on a study where the number of retrieved oocytes in women with progesterone levels > 1.2 ng/ml was 18.2, with a standard deviation of 6.6. The number of retrieved oocytes in women with low progesterone levels was 15.08. Considering alpha 0.05, beta 80%, and an error level of 5%, the required sample size for each group was calculated to be 70, totaling 140 participants.

### Ethical Considerations

This study was approved by the Ethical Committee of Tehran University of Medical Sciences, Tehran, Iran (Code: IR.TUMS.IKHC.REC.1402.519). All participants filled out and signed a written informed consent form.

### Statistical Analysis

We used the Kolmogorov-Smirnov test for normality of data, and after approval normality we used the parametric tests. Descriptive statistical methods, including numbers and percentages and statistical indices such as mean and standard deviation, were employed for data analysis. An independent *t* test was utilized to compare the quantitative variables between the low and normal ORR groups. At the same time, the Chi-square test was applied to compare the qualitative variables between the 2 groups. Additionally, logistic regression analysis was conducted to investigate the variables affecting low ORR. The significance level in all tests was set at < 0.05. The Statistical Package for the Social Sciences, version 24, SPSS Inc., Chicago, Illinois, USA (SPSS) was used for data analysis.

## 3. Results

In this research, 140 women candidates for IVF/ICSI cycles, aged between 18 and 40, were studied. The median BMI was 27.2 kg/m^2^, with an interquartile range of 23.6–29.7 (Table I).

In terms of menstrual cycle pattern, 56 individuals (40%) were classified as oligoamenorrhoea. Regarding ovarian hyperstimulation syndrome status, 37 individuals (26.4%) were categorized as minimal. No significant difference was observed in ovarian hyperstimulation syndrome and menstrual cycle between the 2 groups of women with low and normal ORR (Table II).

ORR ranged from 0.04–0.94 with an average of 0.5 ± 0.23. In the comparison of the quantitative variables between the 2 groups of low ORR and normal ORR women, using the *t* test, a significant difference was observed between the 2 groups only in terms of the variable of estradiol-to-follicle above 17 ratio, follicle more than 17, and number of retrieval oocytes. These variables in women with low ORR group were significantly lower than the women in normal ORR group (Table III).

The results of the logistic regression analysis showed that except for the variable of estradiol-to-follicle above 17 ratios with OR = 0.96 (CI: 0.94–0.98), the other variables had no significant effect on the low ORR index (Table IV).

**Figure 1:**
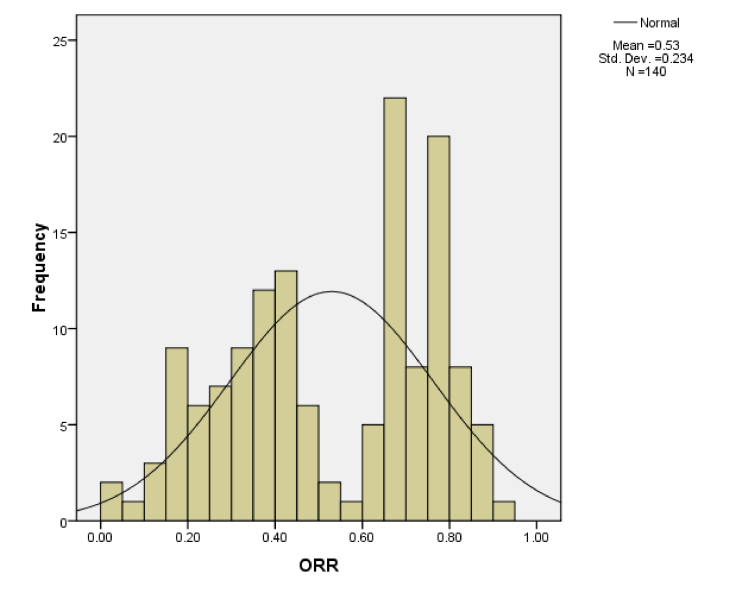
Frequency of oocyte retrieval rate (ORR) values in PCOS cases undergoing IVF/ICSI, with a normal distribution. The ratio of the number of follicles seen on ultrasonography with a diameter of at least 17 mm on the trigger day to the number of oocytes obtained was known as ORR.

Among the available cycles with at least one oocyte retrieved, the distribution of ORR was normal and, according to figure 1, had a median of 0.55, an interquartile range of 0.35–0.74, and maximum and minimum values of 0.04 and 0.94 (Figure 1).

**Table 1:** Descriptive statistics of clinical and demographic variables of studied women

**Variables**	**Mean ± SD**
**Age**	31.8 ± 4.5
**BMI**	26.9 ± 4.3
**Progesterone level (ng/mL)**	1.13 ± 0.5
**Free testosterone level (nmol/L)**	2.2 ± 3.6*
**Estradiol to progesterone ratio (pg/mL)**	3314.5 ± 465.2
**AMH (ng/mL)**	6.3 ± 3.6
**Estradiol to follicles > 17 ratio (pg/mL)**	91.5 ± 1.6
Data presented as Mean ± SD, BMI: Body mass index, AMH: Anti-Mullerian hormone, *IQR: 0.7, MD: 1.5	

**Table 2:** Frequency distribution of variables in studied women

**Variables**		**n (%)**
**Cycles**		
	**Normal**	60 (42.9)
	**Oligoamenorrhoea**	56 (40)
	**Amenorrhoea**	24 (17.1)
**OHSS**		
	**Minimal**	37 (26.4)
	**Mild**	16 (11.4)
	**None**	87 (62.1)
Data presented as n (%), OHSS: Ovarian hyperstimulation syndrome		

**Table 3:** Compare the quantitative indices in studied women in 2 groups

**Variables**	**Groups**		**P-value**
	**Low ORR**	**Normal ORR**	
**Age**	32.3 ± 4.8	31.3 ± 4.1	0.18
**Free testosterone level**	2.7 ± 0.6	1.7 ± 0.1	0.08
**Estradiol to progesterone ratio**	3628.4 ± 904	3000.5 ± 229	0.15
**Estradiol to follicles ≥ 17 ratio**	85.4 ± 15.3	97.6 ± 20.7	< 0.001
**BMI**	27.2 ± 4.3	26.5 ± 4.3	0.36
**AMH**	5.8 ± 2.5	6.8 ± 4.4	0.12
**Progesterone level**	1.15 ± 0.6	1.1 ± 0.5	0.57
**Follicles ≥ 17**	24.03 ± 4.03	30.3 ± 5.1	< 0.001
**Number of retrieval oocytes**	7.7 ± 3.1	22.4 ± 3.9	< 0.001
Data presented as Mean ± SD, Two-sample *t* test. ORR: Oocyte retrieval rate, BMI: Body mass index, AMH: Anti-Mullerian hormone			

**Table 4:** Results of logistic regression to determine the effective variables on low ORR

**Variables**	**Coefficients**	**P-value**	**OR**	**95% CI**
**Age**	-0.06	0.26	0.95	0.86-1.042
**BMI**	0.003	0.96	1.003	0.91-1.11
**Progesterone level**	-0.09	0.83	0.91	0.4-2.1
**Free testosterone level**	-0.24	0.34	0.79	0.48-1.29
**AMH**	0.11	0.16	1.1	0.96-1.3
**Cycles**	0.6	0.37	1.8	0.5-6.9
**OHSS**	-1.26	0.1	0.28	0.06-1.3
**Estradiol to follicle ≥ ratio**	-0.04	0.001	0.96	0.94-0.98
ORR: Oocyte retrieval rate, BMI: Body mass index, AMH: Anti-Mullerian hormone, OHSS: Ovarian hyperstimulation syndrome				

## 4. Discussion

Only the variable of the estradiol-to-follicle ratio above 17 mm was significant among the factors influencing low ORR in this study, according to the results of logistic regression; the other variables had no effect. A similar study found that occurrences with low ORR in PCOS cases can be predicted by the estradiol-to-follicle level ratio of less than 12 mm on the trigger day. The average ORR in this study was 1.02, with a range of 0.6–2.69. In patients with low ORR, the BMI, AFC, gonadotropin stimulation days, progesterone, and estradiol-to-follicle ratio were all higher than those in the normal ORR group. On the day of the trigger, they were less than 12 mm lower.

Logistic regression analysis showed that the variable estradiol-to-follicle above 12 mm ratio, along with the 2 variables AFC and progesterone level on the trigger day, were significantly associated with low ORR, which was in line with our study results.

The optimal cut-off point was 169.2, with a sensitivity of 65% and a specificity of 65% for predicting low ORR, according to the receiver operating characteristic (ROC) curve area, which revealed that the ratio of estradiol to follicle < 12 mm had an area under the ROC curve of 0.7 (11). The ratio of serum estradiol level to the number of follicles ( ≥ 17 mm) seen on ultrasound on the day of the trigger was another predictor of low ORR in this investigation. A frequent hormonal indicator of the ovarian response to stimulation is the serum levels of estradiol which are released by proliferative granulosa cells (12).

The quantity of oocytes recovered has a positive correlation with it (13–14). Peak estradiol levels are thought to be indicative of oocyte maturation, particularly in cycles with few follicles (15). However, the exact estradiol concentration to determine the number or rate of oocyte maturation has not been determined (16–17). This is because estradiol levels vary significantly among instances, even with equivalent ovarian reserve, due to the considerable influence of the number of pre-ovulatory follicles. Since the difference in peak estradiol levels between the 2 groups was not significant and the number of follicles ( ≥ 17 mm in diameter) observed on ultrasound did not significantly alter logistic regression analysis, none of the variables in our study may be used to predict poor ORR.

The current study's regression analysis and intergroup comparison failed to find statistically significant progesterone levels on trigger day. The ROC study indicates that its prognostic influence on the low ORR is less than that of the estradiol/follicle ratio. The granulosa cells of the ovary release progesterone, much like they do estradiol. It has a favorable correlation with the quantity of oocytes and increases in concentration as the follicles mature (18–21). Both high and low amounts of progesterone in the late follicular phase have been shown to decrease the number of oocytes (22). High levels may suggest early ovulation, whereas low levels show inadequate granulosa cell proliferation or malfunction, suggesting that the follicle is not yet nearing maturity.

This conclusion is supported by the results of our investigation. The result of this study confirmed the results of the present study.

A retrospective analysis was conducted on the clinical data of 1254 PCOS cases that satisfied the study's inclusion criteria. They concluded that, in PCOS instances, the clinical pregnancy rate was correlated with progesterone levels, but not with the estradiol to progesterone ratio. In contrast, women in the low ORR and normal groups did not significantly differ in the quality of oocyte retrieval based on the estradiol to progesterone ratio (10). 10,624 oocyte retrieval cycles were split into 2 groups for the study based on whether or not the quantity of oocytes harvested on the day of human chorionic gonadotrophin reached more than 14 mm follicles.

The group with inadequate oocyte retrieval had lower levels of progesterone, estradiol, and luteinizing hormone on the trigger day, the researchers concluded. Similar to this study, women with low ORR had somewhat greater progesterone levels and lower estradiol levels, although the difference was not statistically significant (21). In a retrospective cohort study, 2192 people had a total of 5447 IVF and ICSI cycles evaluated. The researchers concluded that both high and low progesterone levels on the trigger day negatively impacted the live birth rate. Since there was no discernible difference in the progesterone levels between the groups in this study, it is impossible to reach a reliable conclusion in this regard (22).

With a limit of one standard deviation from the mean ORR, the ORR was calculated by splitting the 2478 PCOS cases undergoing ICSI/IVF into 2 groups: low ORR and normal ORR. The researchers found that low ORR, which results in fewer embryos and more cycles being canceled, can be caused by low levels of progesterone on the day of trigger and low levels of estradiol/follicle above 17 mm in PCOS instances. The results of this investigation were similar to those of the current study.

The progesterone hormone level on hCG day and the outcomes of IVF were assessed in a cohort study of 180 Han Chinese women with PCOS who were infertile and undergoing controlled ovarian hyperstimulation using an exogenous gonadotropin/GnRH antagonist protocol. The results of these trials showed that women with greater progesterone levels were more fertile and had higher oocyte retrieval than women with lower progesterone levels; nevertheless, the pregnancy rate did not significantly differ between groups (20). In the current study, progesterone levels were greater in women with low ORR, but there was no appreciable difference between groups.

Due to their detrimental effect on oocyte maturation, high progesterone levels—especially those beyond the established cutoff of 1.54 ng/ml—are a useful clinical predictor of less-than-ideal IVF outcomes, which confirms our study results (23).

### Strengths and limitations

This study's limitation was the small number of participants and the not tending of women to participate in this prospective study due to its time-consuming. The purpose of the present study was to investigate progesterone levels and their relationship with oocyte number. In the study subject, high or low progesterone levels were not considered as a risk factor.

Progesterone levels on the trigger day were lower in the low ORR group than the normal ORR group, although this relationship was not significant. This may be due to the small size of the study population.

## 5. Conclusion

Although this difference was not statistically significant, the low ORR group had lower progesterone levels on the trigger day than the normal ORR group. The limited size of the research population could be the cause of this. Double trigger for oocyte maturation can be considered when comparing the number of oocytes extracted to the typical number and size of follicles in the low ORR group, as well as the lower ratio of estradiol to follicles with a size of 17 mm or greater compared to the normal ORR group. Furthermore, a low dosage of the human chorionic gonadotropin hormone ampoule is advised for PCOS patients.

##  Data Availability

Data supporting the findings of this study are available upon reasonable request from the corresponding author.

##  Author Contributions

N. Bahrami: Study design, review articles, paper preparation, data collection. A. Moini: Review articles, manuscript draft, data collection, clinical examination. L. Kashani: Data collection, review articles, writing results, sampling M. Khodarahmian: Data analysis, review articles, manuscript preparation, data analysis.

##  Conflict of Interest

The authors declare that there is no conflict of interest.
